# Testing a social network approach to promote HIV self-testing and linkage to care among fishermen at Lake Victoria: study protocol for the *Owete* cluster randomized controlled trial

**DOI:** 10.1186/s13063-022-06409-3

**Published:** 2022-06-06

**Authors:** Lila A. Sheira, Zachary A. Kwena, Edwin D. Charlebois, Kawango Agot, Benard Ayieko, Monica Gandhi, Elizabeth A. Bukusi, Harsha Thirumurthy, Carol S. Camlin

**Affiliations:** 1 Division of HIV, Infectious Diseases, and Global Medicine, University of California, San Francisco, 1001 Potrero Ave. Building 100, Ward 84, San Francisco, USA; 2 Centre for Microbiology Research, Kenya Medical Research institute, Nairobi, Kenya; 3 Department of Medicine, Center for AIDS Prevention Studies, University of California, San Francisco, USA; 4 Impact Research and Development Organization, Kisumu, Kenya; 5 Department of Obstetrics, Gynecology and Reproductive Sciences, University of California, San Francisco, San Francisco, USA; 6 Department of Medical Ethics and Health Policy, Perelman School of Medicine, University of Pennsylvania, Philadelphia, USA

**Keywords:** HIV self-testing, Men, PrEP, Urine adherence testing, Social networks, Cluster randomized controlled trial, HIV and human mobility

## Abstract

**Background:**

Nearly 50% of men living with HIV in many countries are unaware of their HIV status; men also have lower uptake of HIV treatment and pre-exposure prophylaxis (PrEP). In SSA, highly mobile men such as those working in fishing communities alongside Lake Victoria have low uptake of HIV testing and low rates of linkage to HIV treatment and PrEP, despite increasing availability of these services. HIV self-testing (HIVST) kits hold promise for overcoming barriers to HIV testing and linkage to services for HIV-positive and HIV-negative men. We describe here a protocol for an HIV status-neutral, social network-based approach to promote HIV testing, linkage to care and prevention, and better health outcomes, including adherence, in fishermen around Lake Victoria.

**Methods:**

Utilizing beach management unit (BMU) registries of fishermen operating in three Lake Victoria fishing communities in Siaya County, Kenya, we completed a census and social network mapping to identify close social networks of men. Network clusters identified by a socially-central lead (“promotor”) and selected to ensure maximal separation between treatment and control will be randomized. Promotors in both arms will receive basic HIV training; intervention promotors are further trained in HIVST to distribute kits to their cluster, while control promotors will distribute to their cluster vouchers for free HIVST at nearby clinics. We will test whether these promoters can enhance linkage to ART and PrEP *after* self-testing, thereby addressing a key limitation of HIVST. We will also measure 6- and 12-month viral load in those living with HIV and PrEP adherence among those without HIV via urine tenofovir levels as objective markers of adherence.

**Discussion:**

This study has the potential to improve HIV health and promote HIV prevention among a hard to reach, at-risk, and highly mobile population of men in Western Kenya—a critical population in Kenya’s HIV prevention and treatment program. Further, if successful, this innovative social networks-based model could be scaled at the regional level to address HIV prevention and care among similarly at-risk populations of men in eastern Africa and inland fisheries settings across the continent.

**Trial registration:**

Self-Test Strategies and Linkage Incentives to Improve ART and PrEP Uptake in Men, registered on February 26, 2021, registration #NCT04772469.

## Introduction

Engagement of men in HIV prevention and treatment is a crucial component of the global response to HIV and AIDS. In sub-Saharan Africa (SSA) in particular, men fall behind their female peers in terms of HIV testing [1], knowledge of HIV status, and once diagnosed, HIV treatment and care outcomes such as adherence [2, 3] and viral load suppression [4]. Taken together, these behaviors and outcomes translate to continued onwards transmission of HIV to women [5].

In Kenya, much of the HIV epidemic is centered in the geographic area surrounding Lake Victoria. Highly mobile men such as those working in fishing communities alongside Lake Victoria have low uptake of HIV testing and low rates of linkage to HIV treatment and PrEP, despite increasing availability of these services [4, 6]. Labor-related mobility [7], transactional sex [8], and the fulfillment of culturally valued masculine roles [9] interact and manifest in vulnerabilities for HIV prevention and treatment. For example, low rates of HIV testing and care engagement among fishermen are influenced by structural factors such as access to services at fixed-location clinics that may be distant from beaches, and regular opening hours for clinics that are not compatible with fishing schedules [10–13]. There also exists a “sex-for-fish” economy (“jaboya”, where fishermen give female traders access to fish in exchange for sex), which contributes to their exceptionally high risks [14–18]. Indeed, a meta-analysis of 44 studies found that 42% of fishermen reported engaging in transactional sex, and of those, nearly half reported unprotected sex [19]. Traditional gender roles implicit to men play a role as well, whereby health-seeking behavior, especially in regard to HIV, can be seen as weak and is intertwined with HIV stigma [20]. These structural and cultural barriers contribute to suboptimal HIV prevention and treatment behaviors among fisherfolk.

HIV self-testing (HIVST) presents an excellent opportunity to bridge these gaps. HIV self-testing kits are a new strategy implemented within the last decade that address concerns about both confidentiality and stigma, as well as reaching vulnerable populations, such as fisherfolk [21]. The World Health Organization supports the use of HIV self-testing kits as an effective and safe means to increase knowledge of one’s HIV status and linkage to HIV care [22]. For those who test negative, HIVST interventions offer a linkage to PrEP, thereby offering available HIV preventative care and reducing subsequent transmission. Studies across SSA show high demand for HIV self-testing kits [23, 24]. In Kenya, a community-based distribution of HIV self-testing kits was found to be highly acceptable and with a substantial completion rate [25].

Providing multiple self-tests to men and encouraging them to distribute tests to men in their social networks (i.e., “secondary distribution”) is a promising but untested strategy to increase men’s testing. Secondary distribution of self-tests within social networks may be a low-cost, efficient way to increase testing coverage, and may also address barriers to accessing clinics during operating hours which can be a barrier for fishermen. Secondary distribution of self-tests by women to their male partners is an effective way to promote partner and couples testing [26, 27]; such approaches are now being implemented in many countries [28]. However, this does not sufficiently leverage the fact that men are highly influenced by their peers. The influence of social networks on health behaviors and outcomes is well established [29–35], and it is known that social network characteristics can influence behavior through the circulation of ideas and social influence [36].

### Objectives and hypotheses

The proposed study aims to identify strategies needed to close gaps in HIV care and prevention cascades in high priority populations and settings in sub-Saharan Africa. These gaps are most pronounced in men, who are less likely than women to test for HIV and seek HIV treatment or prevention services, and in high HIV incidence settings such as western Kenya. We will harness the power of peer influence within men’s close social networks and leverage new technologies such as HIV self-tests and point-of-care PrEP adherence assays to increase men’s HIV testing rates and both measure and support adherence. To this end, the study will test three central hypotheses:

1. A higher rate of HIV testing will be observed after three months among men in networks that receive the intervention compared to control;
2. The intervention will result in higher rates of (a) linkage to ART or PrEP (confirmatory testing and ART referral for positives, and PrEP screening for negatives) and (b) ART and PrEP uptake will be observed within 3 months (+ or – about 1 month) in the intervention group; and
3. Higher rates of virologic suppression will be observed in men living with HIV, and higher PrEP adherence observed among uninfected men, in the intervention group compared to the control group.

## Methods/design

### Trial design

The *Owete* Study is a cluster randomized controlled trial. The unit of randomization is clusters of close social networks of fishermen working on the beaches of Lake Victoria in Siaya County, Kenya.

### Study status

Baseline visits began in September 2021 in the pilot beach and were complete by June 2022. Follow-up visits will occur from April 2022 to July 2022, and study follow-up is estimated to complete by September 2022.

### Study setting

Siaya county borders Lake Victoria and has a population of ~ 1 million [37, 38]. It is divided into six sub-counties of Rarieda, Bondo, Alego Usonga, Gem, Ugenya, and Ugunja, with the beaches in Rarieda and Bondo only. The county has an estimated 79 beaches and nearly 38,000 fisherfolk. Its population relies heavily on fishing and subsistence farming, amidst high rates of poverty. Siaya County is also burdened by high rates of HIV, tuberculosis, and malaria [39, 40]. HIV prevalence is 15.3% [41], 28.9% in those aged 25–49 years [42], and highest among fisherfolk at 32.1%. High-risk behaviors, including concurrent partnerships, the *jaboya* sex-for-fish economy, and low condom use, enhance HIV risks [15, 18].

### Clinical setting

Most clinics in Siaya County are operated by the Kenya Ministry of Health and typically staffed by clinical officers and nurses apart from level 4 or higher facilities that may also have medical officers. HIV and PrEP services are co-located, supported by the United States President’s Emergency for AIDS Relief through local implementing partners and integrated within the clinics. The Siaya sub-counties bordering Lake Victoria have 70 health facilities offering ART with 40,258 patients currently in care. Of the 70 clinics, 23 are within 3.5 km of beach communities. The study will be conducted in partnership with five of these clinics, three of which offer 24 h and weekend services including HIV care and prevention [43]. We will work each health facility in-charge to identify one of their staff to support this study.

#### Eligibility criteria

Eligibility includes being a male aged 18 or older, working as a fisherman or fisheries-related occupation (e.g., net repairers, boat builders, boat mechanics, etc.) who are willing and able to provide informed consent and are not currently participating in another research study related to HIV testing, treatment, and/or prevention. Any individual who is less than 18 years of age, female, lacking adequate cognition to consent or complete the study activities, or involved in another study related to HIV treatment and care will be excluded.

#### Recruitment

All study participants will be recruited using the Beach Management Unit (BMU) registry, which documents the name, contact information, and role of each individual registered to work at a given beach. BMU registration is required by Kenyan law and is thus a comprehensive record for ascertaining all individuals involved with fishing at a given beach.

#### Randomization and treatment allocation

The study team will work with BMU officials to conduct a census/BMU registry update and verification in study communities to identify the population of men eligible for the study. We will screen, recruit, and enroll eligible men who give their informed consent to participate, then measure their close social networks and baseline characteristics. A social network survey will be used to obtain information on the close social networks of fishermen using name-generators. This survey will ask participants to name the men in their community to whom they are most closely connected for domains such as health (whom they would seek health information from or share information with), finances (to whom they would turn to borrow money), and spending time (on work and social activities). Using the social network data obtained from the census along with a name matching algorithm, we will identify close male social networks that exist in study communities. “Close” networks will typically include 4–10 men. The network clusters are selected to have high connectivity to a central leader, who will also act as the intervention promoter, to have multiple overlapping ties within the group, and to have as few ties as possible to other study groups. The network-central “promoter” of HIV testing will be the main individual study staff will contact to implement intervention and control group activities and approached for consent. Local close networks with consenting promoters will be eligible for randomization.

Close social networks will be randomized 1:1 to intervention and control arms using a computer algorithm that performs randomization stratified by beach. All network members will receive the study arm condition to which their network is assigned. Participation of all network members is not a requirement for network randomization. Network/cluster leaders will be invited to participate as promotors. During the promotor training, treatment allocation will be revealed to the promotors and to field staff implementing the training. Given the nature of the intervention, blinding is not possible for study staff nor participants; however, blinding will be maintained for the investigative and data team. There are no plans to remove blinding during the course of the study.

#### Interventions

We aim to enroll and randomize at least 80 clusters across the three beaches. In networks randomized to the intervention, promoters will be given multiple self-tests labeled with a unique network specific number to offer self-tests to men in their close social networks and to motivate them to use the self-tests privately or with their support. The control promoter training will cover general HIV, PrEP, and treatment information, including learning about the benefits of HIV testing, while the intervention promoter training adds training in the usage of oral fluid-based HIV tests and in how to motivate others to use self-tests. After the training, the control promoters will receive vouchers for free HIVST at nearby clinics to distribute to their cluster member while the intervention promoters will receive multiple HIVST kits plus voucher for a small amount of remuneration for distribution to other men. The small remuneration amount in the voucher will be redeemable at participating health facilities when participants go for confirmatory testing. An enhanced control arm was selected over standard of care to permit a comparison of men’s use of HIV self-tests received from other men in their social networks with men’s use of HIV self-tests received in clinics, ensuring that any observed differences were not influenced by receipt of a transport voucher. To equalize the potential influence of a financial incentive on behaviors, men in both arms received vouchers for transport to clinics (for men in control arm, to receive a self-test kit for free from the clinic, and for men in the intervention arm to go to the clinic for confirmatory testing). Promoters in the intervention group will be asked to distribute information and transport vouchers for ART or PrEP when distributing HIV self-tests to men in their close social networks. No stop/discontinuation-criteria have been identified.

### Outcomes

The primary outcome will be self-reported HIV testing in the past 3 months by participants, as measured in the 3-month follow-up surveys. The second primary outcome will be defined as the proportion of individuals who link to ART (if confirmatory HIV testing is positive) or PrEP evaluation (if confirmatory HIV testing is negative), meaning they present at a study facility/clinic (depending on results of HIV testing) within 3 months of the intervention start (defined as from when study promotors receive the kits for distribution).

The third primary outcome is a composite measure meant to capture “success” in HIV prevention and/or treatment behaviors. PrEP adherence will be measured at 6 and 12 months after initiation using our novel technology of *point-of-care rapid testing to assess TFV adherence in urine* (developed by Gandhi in collaboration with Abbott Diagnostics™). The measure will be defined as either adequate levels of tenofovir, indicating PrEP adherence via a point-of-care urine adherence assay (TFV levels of < 1500 ng/mL vs. ≥ 1500 ng/ml to distinguish between inadequate and adequate adherence) among HIV seronegative individuals who screened eligible for PrEP *or* the proportion of HIV-positive participants with viral suppression (defined as HIV RNA < 400 c/mL using the Abbott realTime HIV-1 platform and assay, with a detectable threshold of 40 cells per microliter) at 6 and 12 months.

Finally, as secondary outcomes, we will assess retention on ART or PrEP at 6 and 12 months: Using clinic records, we will assess whether men who initiated PrEP and ART attended scheduled appointments and will compare them to those who were either prescribed PrEP or tested positive for HIV.

#### Provisions for post-trial care

There is no anticipated harm or compensation for trial participation. There are no plans for any post-trial care.

#### Qualitative assessment

A qualitative study will be conducted to explore potential mechanisms of action and to identify barriers and facilitators of the intervention implementation. Qualitative investigations will be focused on three domains (Table 1): (1) mechanisms of action of the intervention that result in success or failure in aim 1 (HIVST usage), aim 2 (linkage to ART or PrEP), and aim 3 (suppressed or unsuppressed or detectable TFV levels in urine), (2) key issues impacting the effectiveness of individual components of the intervention, and (3) barriers and facilitators of implementation of the intervention.

**Table 1:** Qualitative and mixed methods research domains and topics by research aim

Domain	Aim 1 topics	Aim 2 topics	Aim 3 topics
Mechanisms of intervention action	• Attributions for decision to test ◦ Role of promoter: perceived influence ◦ Other barriers and facilitators to testing • Attitudes and expectancies re: use of HIVST • Perceived norms re: testing within close social network • Vicarious efficacy re: HIV self-testing (*seeing peers successfully use HIVST*)	• Attributions for decision to link ◦ Role of promoter: perceived influence ◦Role of incentives • Attitudes and expectancies re: ART and PrEP • Perceived norms re: linkage to ART and PrEP within close social network • Vicarious efficacy re: linkage (*seeing peers successfully link to ART or PrEP*)	• Attributions for ongoing engagement in HIV care (ART) and prevention (PrEP) ◦ Role of promoter: perceived influence ◦ Role of incentives • Perceived norms re: engagement in ART and PrEP within close social network • Vicarious efficacy (*seeing peers successfully engaged in ART or PrEP*)
Factors impacting effectiveness of intervention components	• Knowledge of how to use self-tests (*effectiveness of promoter training*) • Relationship factors (e.g., HIV status disclosure, HIV seroconcordant/discordant status) • Psychological factors (e.g., perceived risk, fear, fatalism, self-efficacy to test for HIV)	• Knowledge of benefits of ART and PrEP (*effectiveness of promoter training*) • Relationship factors (e.g., HIV status disclosure, partner support) • Psychological factors (e.g., expectancies, fear, fatalism, self-efficacy to link)	• Experiences with ART/PrEP (*perceived emotional, physical benefits/costs*) • Effective management of side effects • Relationship factors (e.g., relationship change, disclosure, partner support) • Psychological factors (e.g., ART/PrEP fatigue, changes in risk, self-efficacy)
Barriers and facilitators of implementation	• Role of promoter: salience, trust towards promoter within network • Promoters’ self-perceptions and motivation	• Individual mobility and distance to clinic • Past and current experiences with providers/perceived quality of care	• Individual mobility and distance to clinic • Past and current experiences with providers/perceived quality of care

##### Qualitative study population

1. *In-depth interviews*: up to 40 participants, selected to be equally balanced across study arms (i.e., 20 men per arm)
2. *Key informant interviews*: up to 15 promoters from the intervention, and 10 in the control arm
3. *FGD groups*: 3 focus groups per study arm, with 8–12 participants in each group. FGDs will include participants from all study sites

##### Sampling approach

We will use a qualitative sampling strategy drawing from grounded theory [44], in which sampling, data collection, and analysis processes are iterative: we will use baseline data to define initial sampling categories (balance across study arms, sites and HIV status) for selection of the first several participants in each category and thereafter select additional participants according to new categories based upon emergent findings: sampling is completed when data are ‘saturated’, i.e., no emergent findings elicit a need for additional participants. Thresholds are defined for practical reasons of study schedule and budget.

##### Analytic approach

We will use a rigorous two-phased analysis approach, beginning with (1) qualitative analysis of in-depth and key informant interview and FGD data, using a grounded theoretical approach that involves iterative inductive coding of empirical data (transcripts), followed by a (2) mixed-methods analysis of qualitative data grouped by categories defined using social network survey data, process and fidelity measures, and trial outcome data. This will involve analyzing qualitative data collected within categorized groups of participants including (but not limited to) testers vs. non-testers, linkers vs. non-linkers to ART and PrEP, virally suppressed vs. detectable VL, and PrEP adherent vs. non-adherent. This approach will allow us to identify emergent themes within groupings and to rigorously assess conceptual alignment or differences across groupings, with attention to evidence of contradictory findings and deviant cases in the data.

#### Participant timeline

**Fig. 1:**
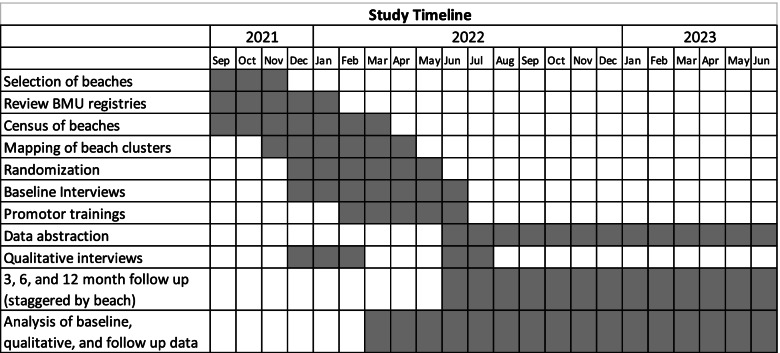
Study timeline

Research staff will collect data from all study participants in both arms at approximately zero months/baseline, 3 months, 6 months, and 12 months (Fig. 1). All assessments will have a window of +/− 1 month. Outcome assessments collected via clinical chart abstraction (HIV confirmatory testing and results, PreP screening and uptake, and ART screening and regimen, clinic attendance), survey (self-reported testing), and via specimen collection and analysis (HIV viral load testing and PreP adherence) will be assessed within 1 month of each participant’s 6- or 12-month outcome window, through a study-scheduled visit.

#### Sample size

**Fig. 2:**
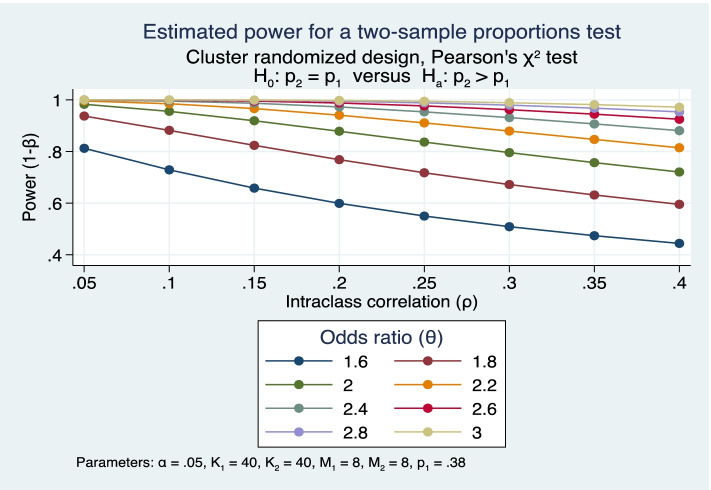
Aim 1 power calculations

We calculate that the study will have an 80% or greater power to detect a significant difference in the proportion of HIV self-testing associated with the intervention arm (aim 1), assuming a proportion self-testing in the control arm of 38% (*observed data from SEARCH study in fishermen*) if the proportion testing in the intervention arm is 52% or greater (odds ratio of 1.8), calculated using an intraclass correlation coefficient (ICC) of 0.15 (*conservative based on SEARCH data*), 40 clusters each in the intervention and control arms, average cluster size of 8 in both arms, and a type-1 error rate of 5% [45, 46].. Figure 2 shows statistical power and the range of detectable odds ratios over extremes of clustering effects.

For aim 2, this study will have a 80% or greater power to detect a significant increase in successful linkage, assuming a proportion successfully linked at 3 months in the control arm of 24% (based on observed SEARCH trial linkage rates) if the observed proportion in the intervention arm is 39% or greater (odds ratio of 2.0), using an ICC of 0.15, 40 clusters each in intervention and control arms, a cluster size of 8 in both arms, and a type-1 error rate of 5%. Statistical power for combined ART or PrEP initiation is similar, with a reduction in anticipated eligible numbers per cluster reduced to 6 resulting in a small increase in the detectable odds ratio to 2.2 or greater. Power for a separate analysis of ART initiation alone is likely to be low given the small numbers of new HIV infections in the study sample with odds ratios in excess of 3 or 4 required.

For aim 3, we will have ≥ 80% power to detect a significant increase in viral suppression and adherence in those on ART and PrEP, respectively, assuming a proportion of successes in the control arm of 70% or greater power. We assume the observed proportion of successes in the intervention arm will be 89% or greater using an ICC of 0.10, a design effect from clustering of 1.7, and a type-1 error rate of 5%. For the combined primary outcome of retention on ART or PrEP at 12 months, this study will have 81% or greater power to detect a significant increase in linkage assuming a proportion retained in care in control arm of 81% if the observed proportion in intervention arm is 96% or greater using the above assumptions.

#### Data collection, management, and safety

Data collected for this study will be entered into handheld computer tablets operating RedCap system. The database will be protected by a separate password on password-protected tablets. Data from the tablets will be uploaded weekly to a UCSF-based server will only be accessible by only necessary study staff. All records will be kept on password protected tablet computers at KEMRI and UCSF. All participant record forms will be kept in individual files in a secured filing cabinet in an access-limited room at the health facility. Participant identifiers will be stripped from the database prior to analysis.

#### Statistical methods

For the test of the intervention effect on the primary outcome of self-reported HIV testing in past 3 months, we will use a cluster adjusted Pearson’s chi-square test comparing the proportion of subjects self-testing in the intervention versus the control arm. In the secondary analysis, we will use multilevel mixed-effects logistic regression with cluster adjusted standard errors and a binary outcome of reported HIV self-testing or not (including cluster as one of the levels with random effects allowed). We will investigate factors that influence the probability of success of HIV self-testing, including characteristics of clusters (the close social networks’ size and composition, metrics of connectedness, mean cluster self-testing rate), characteristics of the cluster’s promoter (age, marital status, HIV status, ART/PrEP status, measures of the promoter’s ties to close-social-network cluster members), and individual level characteristics (demographics, SES, prior HIV testing, self-reported HIV risk, and other health behaviors and characteristics).

For the test of the intervention effect on the primary outcome of linkage to care or prevention within 3 months of intervention start, we will use a cluster adjusted Pearson’s chi-square test comparing the proportion of subjects with documented linkage to care or prevention in the intervention versus the control arm. In the secondary analysis, we will use multilevel mixed-effects logistic regression as described in aim 1 to investigate factors that influence the probability of success of linkage to care and prevention. We will also evaluate the secondary outcomes of ART initiation among HIV-positive persons not currently on treatment, and initiation of PrEP among HIV-negative persons who screen as eligible. We will compare the initiation proportions between study arms using cluster adjusted chi-square tests and additional multilevel mixed-effects logistic regression to identify influencing factors.

We will use a cluster adjusted Pearson’s chi-square test comparing the proportion of successes (HIV viral suppression and adequate TFV levels) among men (on ART and PrEP, respectively) in the intervention and control networks. For the outcome of retention on ART or PrEP at 12 months, we will use a cluster adjusted Pearson’s chi-square test comparing the proportion of subjects on ART or PrEP at 3 months post-trial start who are retained in care at 12 months in intervention versus control group. In the secondary analysis, we will use Cox proportional hazards regression to investigate factors that influence time to discontinuation of ART or PrEP based on clinic visits and chart abstraction. We will also use multilevel mixed-effects logistic regression to investigate factors that influence the probability of viral suppression and TFV detection in urine.

We will define our intent to treat population as those who were randomized to intervention and control groups (regardless of whether they received the kit from their promotor). A secondary, per-protocol analytic group will be defined as those who were members of a cluster that was randomized to the intervention and meet specific criteria per aim will be part of the per-protocol analysis. For aim 1, it will be those individuals in the intervention arm who received an HIV self-testing kit. For aims 2 and 3, it will be those individuals in the intervention arm who received a kit *and* attended a clinic visit.

For the primary intent to treat analysis, we will treat missing outcome variables as failure, i.e., using a failure approach for primary outcomes where the values are treated as zero, not missing. For the per-protocol sample, we will either exclude cases with incomplete data or apply multiple imputation techniques. Analysis of baseline data will occur as beach communities are enrolled and will continue for one calendar year. Final analyses will begin immediately upon completion of all field data collection which is estimated to be completed within twelve months of freezing of the study database. No interim analyses will be conducted.

### Ethics

All study activities have been approved by NIH, the UCSF Institutional Review Board, and the Kenya Medical Research Institute’s Scientific and Ethics Review Unit (KEMRI SERU). Any proposed protocol changes will be submitted to US- and Kenya-based IRBs and will undergo standard review and approval processes and approval at each institute before proposed changes can be implemented. The study has convened an independent and chartered Data and Safety Monitoring Board (DSMB) with HIV research experts based in both Kenya and UCSF who will review and evaluate data related to study enrollment, intervention fidelity, and unexpected issues, including adverse events, on a biannual basis. The DSMB meets biannually to review enrollment, fidelity to intervention, and treatment-arm naïve outcomes. Further, both UCSF and KEMRI will review and renew the study protocol and IRB application on an annual basis.

Study staff will seek informed consent from all participants prior to participation in any study activities. All consent forms will be translated into the local languages and back translated into English to ensure correct use of language. Consent forms will be read aloud to participants by trained staff. Either a signature or a thumbprint (for those who cannot read) in the presence of a witness will be acceptable to confirm informed consent for participation in the study, in the case of written consent forms.

Further, all study participants will be provided with information on how to contact the study staff to report adverse events associated with study participation. Deaths will be reported to the local IRB by email within 48 h after the study team learn of the occurrence and hard copies forwarded to SERU within five working days utilizing standard templates.

## Discussion

Ending the HIV/AIDS epidemic will require novel interventions to seek those hardest to reach for HIV testing and treatment. This upcoming study will address gaps in prevention and care of a highly mobile, hard-to-reach, at-risk population of fishermen working on the beaches of Lake Victoria, Kenya. This study extends the work of previous studies demonstrating that HIV self-testing kits are a safe, acceptable, and even sought after strategy for those who experience barriers to HIV testing [23, 24]. Further, this study combines self-testing with a social-network based approach, an untested but low-cost and potentially highly efficient strategy that capitalizes on strong male social networks. To our knowledge, no other study has tested whether male social networks are an effective strategy for HIV-self-testing utilization. By combining HIV-self-testing with social-network based distribution, our study will provide timely information on strategies for engaging males in HIV prevention and treatment. Moreover, our study will include a unique component of assessing adherence via a point-of-care urine test once linked to PrEP if testing is negative for HIV or assessing the success of HIV treatment if screening is positive.

The primary study team will have access to a fully unblinded database upon conclusion of data collection and preliminary data cleaning is conducted. Study findings will be disseminated in line with both the NIH data dissemination policy and the National Clinical Trials policy. Study findings will be updated on the clinical trials registry at least annually to reflect study enrollment and completion status, with final results uploaded within one calendar year of the completion of data collection. Findings will also be disseminated locally and nationally to key stakeholders. Participant-deidentified data will be made available to interested investigators who propose ancillary analyses who submit a concept sheet proposal to and receive approval from the investigative team; the investigative team will be included as co-authors for all manuscripts resulting from study data.

Upon study conclusion, we will have tested the efficacy of a social-network based intervention to improve access and linkages to HIV testing and care, and prevention, respectively. These findings have the potential to address gaps in the prevention and care of HIV cascade among high-risk for HIV transmission, hard-to-reach populations, such as fishermen. Further, the findings from the qualitative components of the study will address potential scientific and implementation science gaps in using novel technologies, such as HIV self-testing kits, to promote HIV testing and treatment. These study findings will be generalizable to men in fishing communities in other countries that share borders with Lake Victoria, where HIV also concentrates [11, 12, 47–49], in other inland fisheries settings in sub-Saharan Africa [50–53], as well as with translatable applications to other hard-to-reach but high risk of HIV transmission groups.

### Trial status

The *Owete* study is still recruiting participants. Recruitment began in September 2021 and is estimated to be completed in June, 2022. The current protocol number and version is version 9.0 October 27, 2021.

## Data Availability

Interested investigators who propose ancillary analyses can submit a concept sheet proposal to the investigative team.

## References

[1] UNAIDS. THE GAP REPORT, vol. 2014. Geneva: UNAIDS; 2014.

[2] UNAIDS (2014). 90-90-90: An ambitious treatment target to help end the AIDS epidemic.

[3] Boullé C, Kouanfack C, Laborde-Balen G, Boyer S, Aghokeng AF, Carrieri MP (2015). Gender differences in adherence and response to antiretroviral treatment in the Stratall trial in rural district hospitals in Cameroon. J Acquir Immune Defic Syndr.

[4] Maman D, Zeh C, Mukui I, Kirubi B, Masson S, Opolo V (2015). Cascade of HIV care and population viral suppression in a high-burden region of Kenya. AIDS (London, England).

[5] Vandormael A, Akullian A, Siedner M, de Oliveira T, Bärnighausen T, Tanser F (2019). Declines in HIV incidence among men and women in a South African population-based cohort. Nat Commun.

[6] Buchbinder SP, Liu AY (2016). CROI 2016: Hot spots in HIV infection and advances in HIV prevention. Top Antivir Med.

[7] Cassels S (2020). Time, population mobility, and HIV transmission. Lancet HIV.

[8] Naigino R, Wagner GJ, Mukasa B, Musoke W, Sileo KM, Bogart LM (2019). HIV fatalism and engagement in transactional sex among Ugandan fisherfolk living with HIV. SAHARA: Journal of Social Aspects of HIV/AIDS Research Alliance.

[9] Mbonye M, Siu G, Seeley J. Marginal men, respectable masculinity and access to HIV services through intimate relationships with female sex workers in Kampala, Uganda. Soc Sci Med. 2022;296:114742. 10.1016/j.socscimed.2022.114742 35121368

[10] Camlin CS, Ssemmondo E, Chamie G, El Ayadi AM, Kwarisiima D, Sang N (2016). Men “missing” from population-based HIV testing: insights from qualitative research. AIDS Care.

[11] Sileo KM, Wanyenze RK, Kizito W, Reed E, Brodine SK, Chemusto H, et al. Multi-level determinants of clinic attendance and antiretroviral treatment adherence among fishermen living with HIV/AIDS in communities on Lake Victoria, Uganda. AIDS and Behavior. 2019;23(2):406-17. 10.1007/s10461-018-2207-1 PMC6492274 29959718

[12] Seeley JA, Allison EH (2005). HIV/AIDS in fishing communities: challenges to delivering antiretroviral therapy to vulnerable groups. AIDS Care.

[13] Camlin CS, Charlebois ED, Getahun M, Akatukwasa C, Atwine F, Itiakorit H (2020). Pathways for reduction of HIV-related stigma: a model derived from longitudinal qualitative research in Kenya and Uganda. J Int AIDS Soc.

[14] Kwena ZA, Bukusi EA, Ng'ayo MO, Buffardi AL, Nguti R, Richardson B (2010). Prevalence and risk factors for sexually transmitted infections in a high-risk occupational group: the case of fishermen along Lake Victoria in Kisumu. Kenya Int J STD AIDS.

[15] Camlin CS, Kwena ZA, Dworkin SL, Jaboya vs. (2013). jakambi: status, negotiation, and HIV risks among female migrants in the “sex for fish” economy in Nyanza Province, Kenya. AIDS Educ Prev.

[16] Camlin CS, Kwena ZA, Dworkin SL, Cohen CR, Bukusi EA (2014). “She mixes her business”: HIV transmission and acquisition risks among female migrants in western Kenya. Soc Sci Med.

[17] Fiorella KJ, Camlin CS, Salmen CR, Omondi R, Hickey MD, Omollo DO (2015). Transactional fish-for-sex relationships amid declining fish access in Kenya. World Dev.

[18] Kwena ZA, Camlin CS, Shisanya CA, Mwanzo I, Bukusi EA (2013). Short-term mobility and the risk of HIV infection among married couples in the fishing communities along Lake Victoria, Kenya. PLoS One.

[19] Smolak A (2014). A meta-analysis and systematic review of HIV risk behavior among fishermen. AIDS Care.

[20] Siu GE, Wight D, Seeley JA (2014). Masculinity, social context and HIV testing: an ethnographic study of men in Busia district, rural eastern Uganda. BMC Public Health.

[21] Harichund C, Moshabela M (2018). Acceptability of HIV self-testing in sub-Saharan Africa: scoping study. AIDS Behav.

[22] World Health Organization. Guidelines on HIV self-testing and partner notification: supplement to consolidated guidelines on HIV testing services. Geneva: World Health Organization; 2016. 27977094

[23] Mavedzenge SN, Luecke E, Lopez A, Wagner D, Hartmann M, Lutnick A (2016). HIV testing among key populations, adolescent girls and men in Eastern and Southern Africa: a review of research, policy and programming. Methods..

[24] Choko AT, Desmond N, Webb EL, Chavula K, Napierala-Mavedzenge S, Gaydos CA (2011). The uptake and accuracy of oral kits for HIV self-testing in high HIV prevalence setting: a cross-sectional feasibility study in Blantyre, Malawi. PLoS Med.

[25] Wilson KS, Mugo C, Katz DA, Manyeki V, Mungwala C, Otiso L, et al. High acceptance and completion of HIV self-testing among diverse populations of young people in Kenya using a community-based distribution strategy. AIDS and Behavior. 2022;26(3):964-74. 10.1007/s10461-021-03451-1 PMC8409270 34468968

[26] Masters SH, Agot K, Obonyo B, Napierala Mavedzenge S, Maman S, Thirumurthy H (2016). Promoting partner testing and couples testing through secondary distribution of HIV self-tests: a randomized clinical trial. PLoS Med.

[27] Thirumurthy H, Masters S, Napierala Mavedzenge S, Maman S, Omanga E, Agot A (2016). Promoting male partner HIV testing and safer sexual decision making through secondary distribution of self-tests by HIV-negative female sex workers and women receiving antenatal and post-partum care in Kenya: a cohort study. Lancet HIV.

[28] Project Star International (2018). HIV self-testing Africa: the STAR Initiative.

[29] Monge PR, Contractor NS (2003). Theories of communication networks.

[30] Valente TW (2010). Social networks and health: models, methods, and applications.

[31] Newman M, Barabási AL, Watts D (2006). The structure and dynamics of networks.

[32] Wasserman S, Faust K (1994). Social network analysis: methods and applications.

[33] Cross R, Parker A (2006). The hidden power of social networks: understanding how work really gets done in organizations.

[34] Sacerdote B (2001). Peer effects with random assignment: Results for Dartmouth roommates. Q J Econ.

[35] Christakis NA, Fowler JH (2007). The spread of obesity in a large social network over 32 years. N Engl J Med.

[36] Christakis NA, Fowler JH (2009). Social network visualization in epidemiology. Norsk Epidemiol = Norwegian J Epidemiol.

[37] County Government of Siaya (2013). County Integrated Development Plan 2013 – 2017.

[38] County Government of Siaya (2015). County Annual Development Plan 2016-2017.

[39] Wamai RG (2009). The Kenya Health System—analysis of the situation and enduring challenges. JMAJ..

[40] Kes A, Ogwang S, Pande R, Douglas Z, Karuga R, Odhiambo FO (2015). The economic burden of maternal mortality on households: evidence from three sub-counties in rural western Kenya. Reprod Health.

[41] Kenya National AIDS and STI Control Programme. Preliminary KENPHIA 2018 Report. National AIDS and STI Control Program (NASCOP) Accessed 14 Apr 2020.

[42] Kenya National AIDS and STI control programme (2016). Kenya HIV County Profiles.

[43] Kenya Ministry of Health (2013). The state of the health referral system in Kenya: results from a baseline study on the functionality of the health referral system in eight counties.

[44] Charmaz K (2014). Constructing grounded theory.

[45] Ahn C, Heo M, Zhang S (2015). Sample size calculations for clustered and longitudinal outcomes in clinical research.

[46] Campbell MJ, Walters SJ (2014). How to design, analyse and report cluster randomised trials in medicine and health related research.

[47] Kiwanuka N, Ssetaala A, Nalutaaya A, Mpendo J, Wambuzi M, Nanvubya A (2014). High incidence of HIV-1 infection in a general population of fishing communities around Lake Victoria, Uganda. PLoS One.

[48] Kapesa A, Basinda N, Nyanza EC, Mushi MF, Jahanpour O, Ngallaba SE (2018). Prevalence of HIV infection and uptake of HIV/AIDS services among fisherfolk in landing Islands of Lake Victoria, north western Tanzania. BMC Health Serv Res.

[49] Asiki G, Mpendo J, Abaasa A, Agaba C, Nanvubya A, Nielsen L (2011). HIV and syphilis prevalence and associated risk factors among fishing communities of Lake Victoria, Uganda. Sex Transm Infect.

[50] Nagoli J, Holvoet K, Remme M (2010). HIV and AIDS vulnerability in fishing communities in Mangochi district, Malawi. Afr J AIDS Res.

[51] MacPherson EE, Sadalaki J, Njoloma M, Nyongopa V, Nkhwazi L, Mwapasa V (2012). Transactional sex and HIV: understanding the gendered structural drivers of HIV in fishing communities in Southern Malawi. J Int AIDS Soc.

[52] Merten S, Haller T (2007). Culture, changing livelihoods, and HIV/AIDS discourse: reframing the institutionalization of fish-for-sex exchange in the Zambian Kafue Flats. Cult Health Sex.

[53] Béné C, Merten S (2008). Women and fish-for-sex: transactional sex, HIV/AIDS and gender in African fisheries. World Dev.

